# Short-time cycling performance in young elite cyclists: related to maximal aerobic power and not to maximal accumulated oxygen deficit

**DOI:** 10.3389/fphys.2024.1536874

**Published:** 2025-01-10

**Authors:** Eva Maria Støa, Bent Rønnestad, Jan Helgerud, Jan-Michael Johansen, Ingvild Tronstad Andersen, Torkil Rogneflåten, Anders Sørensen, Øyvind Støren

**Affiliations:** ^1^ Department of Sports, Physical Education and Outdoor Studies, University of South-Eastern Norway, Kongsberg, Norway; ^2^ Section for Health and Exercise Physiology, Institute of Public Health and Sport Sciences, Inland Norway University of Applied Sciences, Lillehammer, Norway; ^3^ Department of Circulation and Medical Imaging, Norwegian University of Science and Technology, Trondheim, Norway; ^4^ Myworkout, Medical Rehabilitation Centre, Trondheim, Norway; ^5^ Department of Natural Sciences and Environmental Health, University of South-Eastern Norway, Kongsberg, Norway

**Keywords:** cycling time-trial performance, maximal aerobic power, maximal anaerobic power, oxygen cost of cycling, maximal accumulated oxygen deficit

## Abstract

**Purpose:**

To explore the relationships between performance variables and physiological variables in a short-time (2–3 min) cycling time trial (TT) on a cycle ergometer.

**Methods:**

Fifteen young elite cyclists (age: 17.3 ± 0.7 years, maximal oxygen uptake (VO_2max_): 76.6 ± 5.2 mL⋅kg^−1^⋅min^−1^) participated in this study. Maximal aerobic power (MAP), maximal anaerobic power (MANP), time to exhaustion at 130% of maximal aerobic power (TTE), maximal accumulated oxygen deficit (MAOD) in the TT, anaerobic power reserve (APR) and lactate threshold (LT) was tested. MAP was calculated as VO_2max_/oxygen cost of cycling (C_C_), MANP was determined as mean power output (W) during a 10 s maximal cycling sprint test, and MAOD was calculated as (VO_2_ demand - VO_2_ measured) ∙ time. APR was calculated as the relative difference between MAP and MANP.

**Results:**

There was a strong correlation between MAP and TT time (r = −0.91, p < 0.01) with a standard error of estimate (SEE) of 4.4%, and a moderate association between MANP and TT time (r = −0.47, p = 0.04). Neither MAOD, TTE, LT nor APR correlated with TT.

**Conclusion:**

MAP was highly correlated with TT with a SEE of 4.4%. Since neither TTE nor MAOD correlated with TT, this indicates that these two variables do not play a significant role in differentiating short-time endurance cycling performance. We suggest training for improving MAP and, or MANP to improve short-time endurance cycling performance.

## 1 Introduction

Time performance in short-time cycling (e.g., between 1 and 5 min) put great demands on both the aerobic and anaerobic energy systems (Medbø and Tabata, 1989). To quantify the relations between variables representing these energy systems may therefore provide valuable insights on which variables that may or may not determine time performance. In two previous studies on middle distance running and sprint ergometer skiing (Støren et al., 2021; Støren et al., 2023), both maximal aerobic power (MAP), and maximal anaerobic power (MANP) correlated strongly with short time trial performance. However, in these two studies, the ability to sustain a high anaerobic intensity, measured as either time to exhaustion at 130% MAP or maximal accumulated oxygen deficit (MAOD), was not associated with the short time trial performance. MAP integrates both maximal oxygen uptake (VO_2max_) and oxygen cost of cycling (C_C_) and can be expressed as VO_2max_ divided by C_C_ (Joyner and Coyle, 2008; Støren et al., 2013). VO_2max_ and, or C_C_ are both important determinants of endurance cycling performance (Craig et al., 1993; Faria et al., 2005; Joyner and Coyle, 2008; Støren et al., 2013; Babault et al., 2018). MAP has been shown to be highly predictive of performance in both cycling and middle-distance running (Ingham et al., 2008; Støren et al., 2013; Støren et al., 2021). Studies have also shown a positive relationship between the power output or velocity at lactate threshold (LT_W_ or LT_V_) and aerobic endurance performance (Bentley et al., 2001; Faria et al., 2005; Støa et al., 2020). On the other hand, several studies have not found any relationship between LT expressed as percentage of VO_2max_ (LT%) and aerobic endurance performance (McLaughlin et al., 2010; Støren et al., 2013; Sunde et al., 2019; Støa et al., 2020).

MANP expressed as power (W) represents the highest power output achievable during a sprint, and can be measured as the peak power during a short-time all out test (Smith et al., 2001; Faria et al., 2005; Driss and Vandewalle, 2013). To our knowledge, no studies have evaluated the relationship between MANP measured during a 10 s sprint and performance in cycling with a time span like middle-distance running (2–3 min), equal to ∼ 3,000 m individual pursuit track cycling. In studies of sprint skiing, however, higher maximal sprint velocities have been observed in skiers with higher performance levels (Stöggl et al., 2015; Sandbakk et al., 2011; Støren et al., 2023). The same relationship has been indicated in middle-distance runners (Bachero-Mena et al., 2017; Støren et al., 2021). While MANP denotes the absolute peak power output, MAOD represents a volume of anaerobic work over a given time.

Studies from cycling, running and cross-country skiing indicate MAOD to be higher for specialized sprinters and middle-distance athletes than for long distance athletes (Scott et al., 1991; Gastin and Lawson, 1994; Losnegard and Hallén, 2014). However, the role of MAOD in middle-distance performance is equivocal. Craig et al. (1993) found a negative correlation between time performance in 4,000 m cycling and MAOD in a 5 min protocol, while no correlation was found using a 2 min protocol. Billat et al. (2009) and Losnegard et al. (2012) also found a negative correlation between MAOD and 800 m running time performance and 600 m sprint skiing respectively. In contrast to this, Craig and Morgan (1998) found no relationship between MAOD and 800 m running performance. Accordingly, Støren et al. (2023) found no relationship between MAOD and time performance in double poling ergometer sprint skiing.

In sports like running, velocity rather than power output is used for describing maximal aerobic speed (MAS) and maximal anaerobic speed (MANS). The same applies for the anaerobic sprint reserve (ASR), which is defined as the difference between MAS and MANS. To reduce the use of abbreviations in this article, only MAP, MANP and APR are used, also when referring to MAS, MANS and ASR. Application of these terms can be advantageous, since these denotations are the same as in the performance tests, i.e., km·h^−1^, m·min^−1^ or watt. Exploring these variables may therefore be a valuable tool when developing optimal training programs.

Time to exhaustion (TTE) at a velocity or power output exceeding MAP has been used as a surrogate measure of anaerobic capacity and is often tested at an intensity between 120- and 140%VO_2max_ (Blondel et al., 2001; Støren et al., 2021). Previous research has reported strong correlations between TTE and APR (Blondel et al., 2001; Støren et al., 2021; Støren et al., 2023). Regarding a possible impact of TTE and APR on middle-distance performance, Støren et al. (2021) reported no correlation between neither TTE nor APR on 800 m running time performance, nor did Støren et al. (2023) in double poling ergometer sprint skiing. Sandford et al. (2019a) found a strong relationship between APR and 800 m running performance, while this was not the case in 1,500 running performance (Sandford et al., 2019b). To the best of our knowledge no studies have assessed the impact of APR in short-time performance (2–3 min) in elite cyclists. Accordingly, no previous studies have concurrently investigated the impact of MAP, MANP, LT, TTE, MAOD and APR on performance in short time cycling performance. This may also enhance our knowledge of which variables to focus on for improvements of time performance in shorter durations. Although relationships alone do not represent causality, they may serve as an important base for future interventions.

The research question of this study was therefore:

What are the relationships between MAP, MANP, TTE, MAOD, APR and LT, and cycling performance in a short-time ergometer time trial representing ∼ 3,000 m individual pursuit track cycling ? Based on the two previous studies with a very similar design, on running (Støren et al., 2021) and sprint ergometer skiing (Støren et al., 2023), the expected outcome was that MAP, LTw, and MANP would correlate significantly with TT, while MAOD and TTE and LT% would not.

## 2 Materials and methods

### 2.1 Subjects

Fifteen junior (17.3 ± 0.7 years) male elite road cyclists participated in this cross-sectional study. The cyclists were recruited by invitation to the regional top sport high - school, with the selection criteria of being competitive cyclists at a high national level, and with a VO_2max_ ˃ 65 mL⋅min^−1^⋅kg^−1^. The participants were tested in the beginning of the competition period at Inland Norway University of Applied Sciences (INN). Average VO_2max_ was 76.6 ± 5.2 mL⋅min^−1^⋅kg^−1^. Subject characteristics are shown in Table 1. All participants gave written informed consents to participate in the study after oral and written information about the purpose and content of the study. The study was approved by Norwegian Centre for Research Data (183455), and the institutional research board at University of South-Eastern Norway. All the procedures undertaken in the study is in accordance with the principles outlined in the Declaration of Helsinki.

**TABLE 1:** Cyclists characteristics and results (N = 15).

Subject characteristics		
Age (years)	17.3 ± 0.7	(4.0)
BW (kg)	68.8 ± 6.1	(8.9)
Height (cm)	179.7 ± 4.8	(2.7)

Physiological capacities		
VO_2max_		
mL⋅min^−1^⋅kg^−1^	76.6 ± 5.2	(6.8)
C_C_		
mL⋅kg^−1^⋅W^−1^	0.208 ± 0.019	(9.1)
MAP		
W^−1^	370.2 ± 32.2	(8.7)
MANP		
W^−1^	1,005.9 ± 101.0	(10.0)
APR		
%MAP	272.7 ± 26.9	(9.9)
W	635.7 ± 91.6	(14.4)
LT		
[La^−1^]_b_ ^a^	3.6 ± 0.3	(8.3)
W^a^	278.9 ± 42.0	(15.1)
%VO_2max_ ^a^	75.3 ± 9.0	(12.0)
MAOD		
mL⋅kg^−1^	90.8 ± 11.8	(13.0)

Performance variables		
TTE		
S	103.6 ± 24.9	(24.0)
W	509.5 ± 50.6	(9.9)
[La^−1^]_b_	11.6 ± 2.6	(22.4)
TT		
S	158.2 ± 16.8	(11.0)
W	481.2 ± 51.2	(10.6)
[La^−1^]_b_	13.6 ± 2.3	(16.9)

Results are presented as mean ± standard deviation, with the coefficient of variance in percentage in parenthesis. BW, body weight; Kg, kilograms; Cm, centimeters; VO_2max_, maximal oxygen uptake; mL⋅min^−1^⋅kg^−1^, milliliters per kg body mass per minute; C_C_, oxygen cost of cycling; mL⋅kg^−1^⋅W^−1^, milliliters per kg body mass per watt; W, watt; MAP, maximal aerobic power; MANP, maximal anaerobic power; APR, anaerobic power reserve, i.e., the difference between MAP and MANP or in per cent of MAP; LT, lactate threshold; S, seconds; TTE, time to exhaustion at 130% MAP; [La^−1^]_b_, blood lactate concentration in millimole; MAOD, mean accumulated oxygen deficit; mL⋅kg^−1^, milliliters per kg body mass; TT, time trial.

^a^ N = 14.

### 2.2 Design

This study is part of a larger project investigating variables relevant to performance in middle-distance events across three different sports: cross-country skiing, running, and cycling. The methods described here therefore align closely with previously published articles within this project (Støren et al., 2021; Støren et al., 2023).

The present study is a cross-sectional study exploring the relationships between physiological variables and short-time (2–3 min) cycling time trial (TT) in a laboratory setting.

### 2.3 Testing

**FIGURE 1:**
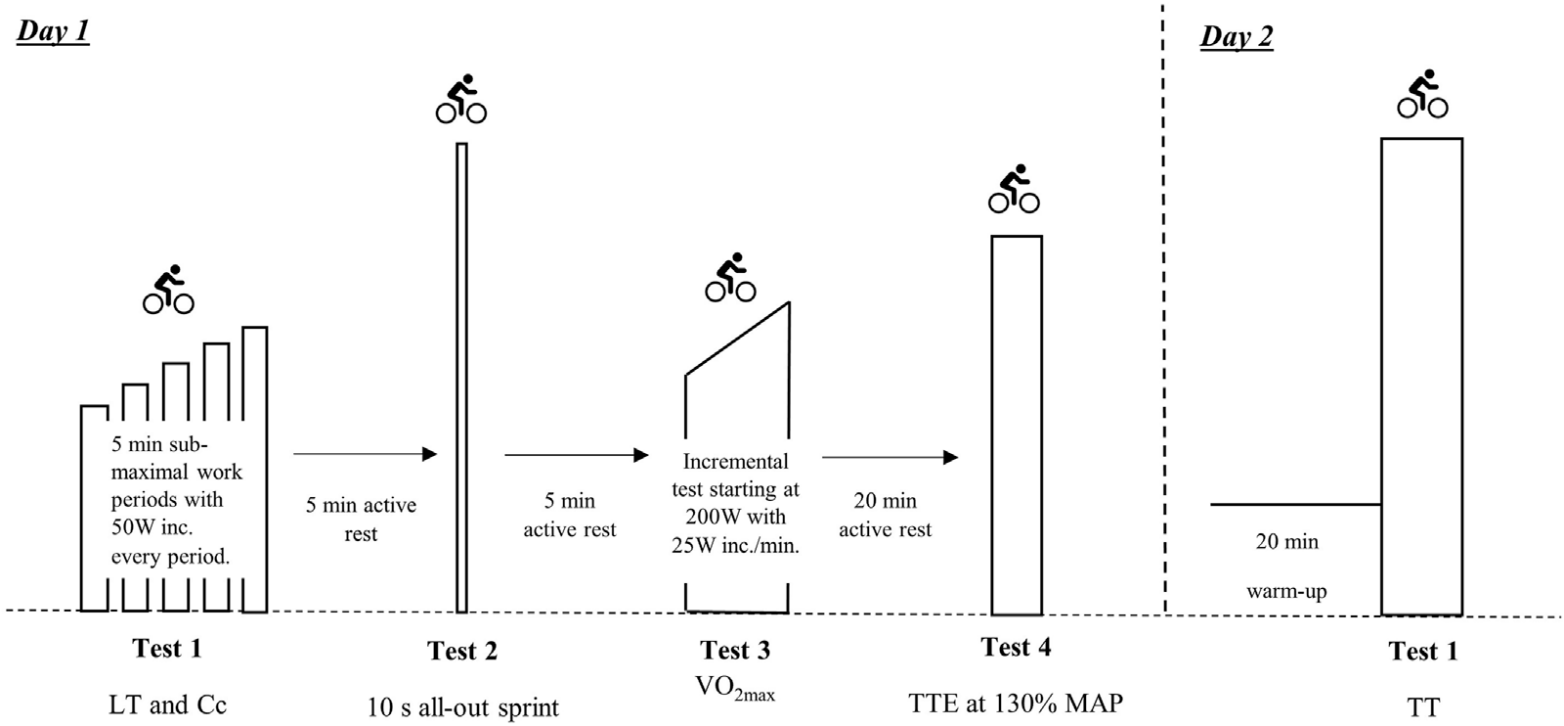
Test protocol. LT, lactate threshold. C_C_, oxygen cost of cycling. S, seconds. W, watt. VO_2max_, maximal oxygen uptake. TTE, time to exhaustion at 130% MAP. MAP, maximal aerobic power. TT, time trial.

The participants were tested on two occasions separated by 5–10 days. Both test days were performed at the same laboratory, with the same temperature of 19° ± 1° celcius. The cyclists were familiarized with the test bikes with a light submaximal warm up preceding the first test at day one. The familiarization also included individual fitting of the test bike. Being junior elite cyclists, all participants were familiar with indoor stationary cycling. The test protocol is presented in Figure 1. The participants were instructed not to perform any strenuous physical training the last 24 h before the test days. Body weight, body height, LT, C_C_, 10 s sprint, VO_2max_ and TTE were tested on the first test day. On the second day, the cycling TT was performed on the test bike, at a set distance representing approximately 3,000 m individual pursuit track cycling. Continuous VO_2_ measurements to assess MAOD, was tested during the TT. All physical tests were carried out using a Lode Excalibur Sport ergometer (Lode B. V., Groningen, Netherlands) and VO_2_ was measured using a computerized metabolic system with mixing chamber (Oxycon Pro, Erich Jaeger, Hoechberg, Germany). The gas analyzers were calibrated using certified calibration gases of known concentrations before each test. The flow turbine (Triple V, Erich Jaeger, Hoechberg, Germany) was calibrated using a 3 L, 5,530 series, calibration syringe (Hans Rudolph, Kansas City, United States) before each test.

The first test day started with a blood lactate profile test that was initiated with 5-minute cycling at 125 W and increased by 50 W every fifth minute [25 W if (La^−^) was ≥3 mmol·L^−1^] and terminating when [La^−^] reached ≥4 mmol·L^−1^. Blood samples were taken from a fingertip at the last 30 s of each 5-minute bout, being immediately analyzed (Biosen C-Line, EKF Diagnostics, Penarth, United Kingdom). VO_2_ was measured during the last 3 min of each bout. Power output at 4 mmol·L^−1^ [La^−^] was calculated from the relationship between [La^−^] and power output in the last two stages, by using linear regression. C_C_ was calculated as the O_2_ cost at the nearest actual power representing approximately 70% MAP. Following the LT test, cyclists pedaled for 5 min at a power output between 50 and 100 W before the 10-s sprint was performed. The sprint was performed in a seated position. Following a 3-second countdown, braking resistance (0.85 Nm·kg^−1^ body mass) was applied to the flywheel and remained constant throughout the 10-second sprint test. This braking resistance was chosen according to manufacturer’s guidelines and pilot testing at the laboratory. Participants remained seated throughout the sprint and were given strong verbal encouragement. At the signal “Go,” the participants were instructed to pedal as fast as possible from the start. After a 5-min recovery period, a maximal incremental test to determine VO_2max_ and MAP was performed. The test started at 200 W, with work-rate being increased by 25 W every minute until volitional exhaustion, or cyclist’s inability to maintain cadence above 60 rounds per minute (rpm) despite verbal encouragement. Cadence was freely chosen. Pulmonary gas exchange was continuously measured, and VO_2max_ was calculated as the highest 60-second mean oxygen uptake. MAP was calculated as VO_2max_/C_C_, as previously described (Helgerud et al., 2010; Sunde et al., 2019; Støa et al., 2020; Støren et al., 2021). After the VO_2max_ test, a 20-minute recovery period was given before TTE at 130% MAP was performed. An intensity relative to MAP for the TTE test was chosen in order to apply the same relative amount of extra anaerobic work in each cyclist. An intensity relative to APR would imply different levels of extra anaerobic work dependent on the size of the APR. 130% MAP was chosen based on the previous work by Blondel et al. (2001) and Støren et al. (2021). This intensity is high enough to ensure supramaximal work relative to VO_2max_, and low enough to ensure a minimum test duration. Based on previous research (Blondel et al., 2001; Støren et al., 2021; Støren et al., 2023), this intensity has resulted in a mean work time of ∼90 s, with a range of ∼30–180 s. In these cyclists, the 130% MAP represented a mean of 17.9% of the APR, with a coefficient of variance of 21.1%. The TTE test was performed until volitional exhaustion, or cyclist’s inability to maintain cadence above 60 rpm despite verbal encouragement.

The second test day was initiated by a 20-min warm-up. After the warm-up, the TT was performed with verbal encouragement.

MAOD was tested during the TT and calculated as the mean difference between VO_2_ demand and measured VO_2_ in the TT. MAOD was expressed as the product of this difference and time (mL⋅kg^−1^). VO_2_ demand was calculated as the product of mean TT power and C_C_, i.e., W ⋅ C (Billat et al., 2009).

Heart rate was registered continuously during all physical tests using a Polar S610i heart rate monitor (Polar, Kempele, Finland).

### 2.4 Statistical analysis

The TT and TTE data were found to be normally distributed by the Shapiro-Wilk test (p = 0.91 and 0.33, respectively) and Q-Q plots. Therefore, the descriptive data are presented as mean ± standard deviation and coefficient of variance (CV). The sample size was determined from the statistical power calculations with numbers from Støren et al. (2013), showing that at least 10 cyclists were needed to obtain a power above 80% and p < 0.5 for the correlation between MAP and TT. In the present study. Pearson bivariate correlational tests were used to assess relationships between selected physiological variables and TT performance and TTE performance. Although the multiple regression analysis was underpowered, they are included in this study. The multiple regressions had TT as dependent variable, and MAP and MANP, or MAP, MANP and MAOD, or MAP and APR as independent variables. These tests were performed since VIF did not indicate co-linearity. The multiple regressions used should, however, be interpreted with caution, since two independent variables calls for at least 30 participants. These regressions were still included to show that MAP and MANP alone gave the highest r-value related to TT. The correlations are displayed by the correlation coefficient r, standard error of estimate (SEE) and for the multiple regressions, also by the variation inflation factor (VIF). For main findings, 95% confidence interval (CI) and Cohens’d are presented in the text. For evaluation of statistically significant correlations, r > 0.7 was strong, r between 0.5 and 0.7 was moderate, and r < 0.5 was week. All statistical analysis were performed by SPSS version 29.1, and the significance level was set as *p* < 0.05 in two-tailed tests.

## 3 Results

The participants’ characteristics and results of VO_2max_, C_C_, MAP, MANP, APR, LT, TTE, MAOD and TT are presented in Table 1.

**FIGURE 2:**
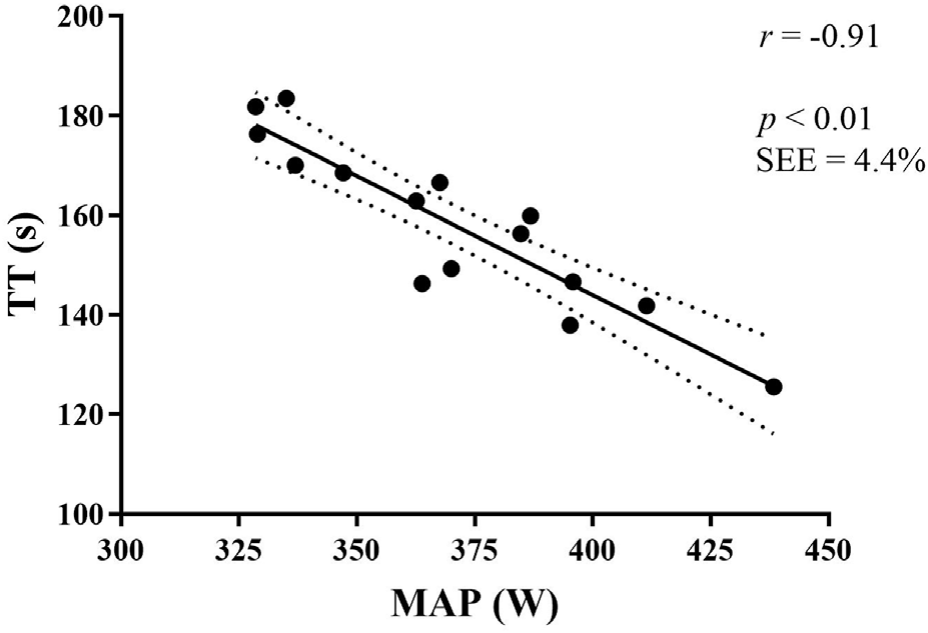
Correlation between MAP and TT performance. TT, time trial. S, seconds. MAP, maximal aerobic power. W, watt.

Correlations with TT performance are presented in Table 2; Figure 2. MAP correlated strongly with TT (r = −0.91, CI −0.97, −0.75, d = 4.33). VO_2max_ (L∙min^−1^) (r = −0.92, CI -0.97, −0.78, d = 4.63) and C_C_ (mL⋅kg^−1^⋅W^−1^) (r = 0.60, CI 0.13, 0.85, d = 1.53) separately both correlated with TT. MANP showed a moderate correlation with TT (r = −0.47, CI −0.79, 0.57, d = 1.06). A multiple regression between TT and MAP∙ MANP resulted a strong correlation (r = −0.92, CI −0.93, 0.48, d = 4.63). Neither TTE, MAOD, APR nor LT correlated significantly with TT.

**TABLE 2:** Correlations with TT performance (N = 15).

	R	P	SEE (%)	VIF
VO_2max_				
L⋅min^−1^	−0.92	<0.01	4.2	
mL⋅kg^−1^⋅min^−1^	−0.34	0.22	10.4	
C_C_				
mL⋅kg^−1^⋅W^−1^	0.60	0.02	8.9	
MAP				
W^−1^	−0.91	<0.01	4.4	
MANP				
W^−1^	−0.47	0.04	9.7	
TTE				
S	−0.06	0.41	11.1	
MAOD				
mL⋅kg^−1^	−0.10	0.72	11.0	
APR				
%MAP	0.34	0.11	10.4	
LT				
%VO_2max_ ^a^	0.42	0.07	10.4	
W^a^	−0.18	0.27	11.3	
Multiple regressions				
MAP ⋅ MANP	−0.92	<0.01	6.7	1.24
MAP ⋅ MANP ⋅ MAOD	−0.82	<0.01	6.3	1.00
MAP ⋅ APR	−0.91	<0.01	6.7	1.23

Results are presented as the correlation coefficient r, the level of significance p, the standard error of the estimate SEE in per cent, and the variation inflation factor VIF as a measure of co-linearity in the multiple regressions. TT, time trial in seconds; MAP, maximal aerobic power; W, watt; MANP, maximal anaerobic power; MAOD, mean accumulated oxygen deficit; mL⋅kg^−1^, milliliters per kg body mass in the TTE test; LT, lactate threshold; TTE, time to exhaustion at 130%MAP; APR, anaerobic power reserve, i.e., the difference between MAP and MANP or in per cent of MAP; LT, lactate threshold; S, seconds; TTE, time to exhaustion at 130% MAP; [La^−1^]_b_, blood lactate concentration.

^a^ N = 14.

Correlations with TTE performance are presented in Table 3. The only single variable which correlated with TTE performance was MAOD (r = 0.77, CI 0.42, 0.92, d = 2.40).

**TABLE 3:** Correlations with TTE performance (N = 15).

	r	P	SEE (%)	VIF
MAP				
W^−1^	−0.26	0.17	15.8	
MANP				
W^−1^	0.06	0.41	16.5	
MAOD				
mL⋅kg^−1^	0.77	<0.01	10.4	
APR				
%MAP	0.28	0.16	15.8	
LT				
%VO_2max_ ^a^	0.21	0.23	16.4	
W^♯^	−0,08	0.39	16.6	
Multiple regressions				
MAP ⋅ MANP	0.33	0.11	16.1	1.24
MAP ⋅ MANP ⋅ MAOD	0.86	<0.01	9.1	1.25
MAP ⋅ APR	0.32	0.11	16.1	1.23

Results are presented as the correlation coefficient r, the level of significance p, the standard error of the estimate SEE in per cent, and the variation inflation factor VIF as a measure of co-linearity in the multiple regressions. TTE, time to exhaustion at 130% MAP in seconds; MAP, maximal aerobic power; W, watt; MANP, maximal anaerobic power; MAOD, mean accumulated oxygen deficit; mL⋅kg^−1^, milliliters per kg body mass in the TTE test; LT, lactate threshold; APR, anaerobic power reserve, i.e., the difference between MAP and MANP or in per cent of MAP; LT, lactate threshold; S, seconds; TTE, time to exhaustion at 130% MAP; [La^−1^]_b_, blood lactate concentration.

^a^ N = 14.

## 4 Discussion

The main findings of the present study were that MAP and MANP showed a strong, and a moderate correlation respectively with TT performance. Neither LT, LT_W_, MAOD, TTE, nor APR correlated with TT performance.

Only a few of these variables have previously been examined within the same study in middle distance events. Since the recent work by Støren et al. (2021), Støren et al. (2023) originates from the same research project as the present study, and investigates all these variables across three different middle distance events, the results from the present study will be compared with their findings. To our knowledge, no other studies have investigated all these variables simultaneously in one study, and we were not able to find any other study investigating the importance of MAP and MANP in shorter cycling TT lasting 2–3 min. In the work of Støren et al. (2021), Støren et al. (2023), negative correlations were observed between MAP and MANP, and 800 m TT in middle-distance running and in ski ergometer double poling. The TT duration in the latter studies ranged ∼120–200 s, i.e., in the same area as the TT time in the present study (158 s), while MANP was measured as average speed and the maximal power respectively during a 100 m sprint (test duration of ∼13 s, and ∼20 s respectively). The present study and the studies of Støren et al. (2021), Støren et al. (2023) represent three different sports, which may account for some small differences in results. In addition, the participants in the present study were young elite athletes. The participants in Støren et al. (2021), Støren et al. (2023) were at a lower mean performance level, but with larger heterogeneity (from recreational to elite). One of the few studies on short-time endurance performance in cycling is Craig et al. (1993), who showed a strong correlation between VO_2max_ and 4,000 m cycling TT. VO_2max_ in the present study showed a strong correlation with TT (r = −0.92). This is also in accordance with Babault el al. (2018) who showed a strong correlation between VO_2max_ and a time trial lasting the same as the present study (103 s). A correlation between C_C_ and TT was found in the present study but not in Craig et al. (1993). MAP, which has incorporated both VO_2max_ and C_C_ did not increase the VO_2max_ correlation with TT in the present study. When aerobic endurance is expressed as MAP, there is no need for allometric scaling since the denotations are divided by body mass both above and below the fractional line. The subjects in the present study were distance cyclists, and not sprint or track endurance specialists as in Craig et al. (1993). It is therefore possible that different physical characteristics could be a reason for the different results. As the product of MAP and LT% in previous studies (Støren et al., 2014; Støa et al., 2020; Johansen et al., 2022) has been shown to determine LTw, one might assume a relationship between LTw and TT in the present study. However, this was not the case, possibly due to the relatively short performance time, implying that the best TT cyclists in the present study were not the cyclists with the highest LTw. This further implies that those with the highest MAP in the present study, did not have the highest LT in % of VO_2max_.

No relationship was found between neither MAOD, TTE nor APR on TT performance. The lack of relationship between MAOD and TT performance in the present study agrees with Craig and Morgan (1998) and Støren et al. (2023) where no correlations were found between MAOD and 800 m TT. However, it contrasts with other studies indicating MAOD to be a determining variable in middle-distance performance (Craig et al., 1993; Billat et al., 2009; Losnegard et al., 2012; Losnegard and Hallén, 2014). The lack of impact of TTE and APR on TT in the present study, is in line with Støren et al. (2021), Støren et al. (2023) who also measured TTE at 130% of MAP. However, due to the lack of studies investigating the importance of MAOD, TTE and APR in short TT cycling performance, further research is required to assess the relative importance of these variables. The use of oxygen debt as an accurate measurement of anaerobic capacity has also been questioned due to the complexity regarding distinct yet closely integrated processes between the anaerobic and aerobic energy systems (Vandewalle et al., 1987; Støren et al., 2021). Not surprisingly, there was a strong correlation between MAOD and TTE in the present study. Both variables may therefore be seen as a measure of anaerobic capacity. This relationship between TTE and MAOD was also observed in Støren et al. (2023).

Despite previous observations of positive correlations between APR and TTE (Støren et al., 2021; Støren et al., 2023), this was not the case in the present study. This could be due to an almost twice as high variation coefficient in TTE in the previous studies compared to the present study (∼45–60% versus 24% respectively). The importance of APR on TT performance has been addressed in other studies (Sandford et al., 2019a; Sandford et al., 2019b; Støren et al., 2021) and is probably dependent on the participants characteristics. For example, Sandford et al. (2019a) found a strong correlation between the corresponding ASR and 800TT among runners with a high MANP in relation to MAP, while this was not the case in a later study, also by Sandford et al. (2019b) among runners competing in 1,500 m. Due to the distinctiveness of the 1,500 m race, the athletes will naturally have a higher MAP, and a lower MANP compared to 800 m specialized runners, and accordingly a smaller APR. This entails that a high APR is positive for middle-distance performance, only if both MANP and MAP are high. If APR is high due to a low MAP, it may not correlate with time performance, as shown in Støren et al. (2021), Støren et al. (2023).

### 4.1 Limitations

There are several limitations in the present study. First, there were only 15 male cyclists participating in the study, and they were not specialized in middle distance cycling events such as track cycling. This reduces the possibility to generalize the results from the present study to other relevant populations. In addition, the cross-sectional design entails a lack of causality regarding the relationships found in the present study and TT performance. These limitations, together with the lack of research exploring these relationships simultaneously in middle distance cycling calls for more future studies. To address causality, intervention studies aiming to improve either MAP, MANP, TTE or MAOD should be performed, and analyzed according to possible improvements in time performance.

### 4.2 Practical applications

A model which can determine the relative contribution of MAP and MANP to short lasting cycling performance, may be of benefit to coaches when developing specific individual training plans for their athletes. MAP and MANP can be measured using only three tests, which will put less strain on both athletes and coaches and could give specific guidelines about what capacity needs to be improved the most. The present study points to the large importance of MAP in young elite level endurance trained cyclists in a short-time endurance event. There are some implications from the present study if the results are representative for elite cyclists in older age categories and if a causality between these correlations and time performance exists. Cyclists competing in corresponding distances or, e.g., want to emphasize a good finish lasting 2–3 min should focus on improving MAP by increasing VO_2max_, e.g., by doing high-intensity aerobic interval training (Rønnestad et al., 2021). Several previous studies (e.g., Helgerud et al., 2007) have shown training intensity between 90% and 95% of VO_2max_ to be effective. Higher intensity training, such as 100% MAP may also improve VO_2max_, as shown by Rhibi et al. (2022). By implementing maximal strength training to improve C_C_ (Sunde et al., 2010), this could also have positive effects on MANP (Kristoffersen et al., 2019). With the low relative importance of TTE and MAOD, the present results suggest to not spend a significant amount of training time on longer supramaximal work periods (e.g., 1 min all out efforts). This is also a very demanding type of exercise, which may cause a long recovery time and therefore reduce the amount of time spent on more effective training.

## 5 Conclusion

Both MAP and MANP showed significant relationships with a short-time TT performance in young elite cyclists, where MAP showed the strongest correlation and the lowest SEE. This highlights the importance of both aerobic endurance and maximal sprint power in short-time cycling TT. In contrast, no relationships were observed between TT and MAOD, TTE or APR.

## Data Availability

The raw data supporting the conclusions of this article will be made available by the authors, without undue reservation.
